# Preparation and characterization of epicuticular wax films

**DOI:** 10.1016/j.heliyon.2019.e01319

**Published:** 2019-03-08

**Authors:** Marco Antônio de Carvalho Faria, Marcos da Silva Sousa, Kevin Figueiredo dos Santos, Nara C. de Souza, Josmary R. Silva

**Affiliations:** Grupo de Materiais Nanoestruturados, Universidade Federal de Mato Grosso, Barra do Garças, Mato Grosso, Brazil

**Keywords:** Materials science

## Abstract

Dipping films from epicuticular wax (EW) were prepared as model systems of epicuticular wax films found in plants. In these films, the growth uniformity, surface morphology, and hydrophobicity were examined. It was observed growth uniformity (linear growth) only from the fifth layer onwards because of the influence of substrate. The surface morphology of the films was found to be composed of pores formed by aggregates of EW molecules, both with a fractal form. An increase in the number of film layers resulted in the increase of the number of pores up to a maximum value followed by a decrease. Such increase was assigned to the growth of aggregates whereas the decrease was explained by the increase of pore sizes, because during the growth of the aggregates, the small pores are replaced by the large pores. Hydrophobicity increased with the number of layers, which was associated with the increase of irregularities on the surface caused by the pores and aggregates. In addition, it was observed that the number of pores increased with temperature. This was explained by the increase in the mobility of EW molecules, which led to a larger amount of EW molecules deposited. Based on our results and the advantages offered by dipping films – including the control of thickness and structure – this type of film is feasible as a model for studies of cuticular water transport in plants.

## 1 Introduction

Epicuticular wax (EW) film plays an important role in controlling the cuticular water transport of plants [1], which is essential to their survival and in the area of food conservation [2]. In addition, these films are protection barriers against microorganisms such as fungi and bacteria [3]. Many types of EW films can be found in nature, which exhibit different chemical compositions and structures [4]. In general, EW films are composed of mixtures of C_20_–C_40_ n-alcohols, n-aldehydes, very long chain fatty acids, and n-alkanes [3]. Such films have thicknesses ranging from the nano- to the micrometer scale [5], and can have crystalline structures [6]. In particular, EW films from grapes have been studied with a focus on their water diffusive transport, which is important for their conservation and drying [7]. The protection against pathogens, such as *Botrytis cinerea*, has also been explored [8]. In addition, the formation, shape and modification of these types of wax have been investigated [9]. EW films of plants exhibit surface morphologies formed by pores [10] and present hydrophobicity. These properties can contribute to the cuticular water permeability, which then controls their water transport phenomenon.

Even though there are studies on EW films *in vivo*, this approach is not convenient for applications of conventional characterization techniques. Sometimes, it is impossible to realize a controlled experiment. To overcome this drawback, a strategy is to use reconstituted epicuticular wax (REW) films as models for EW films [10]. Using REW films, it is possible to examine several properties exhibited by EW films, such as crystalline structure [6], diffusion of water [1], surface morphology [4, 11], and wettability [2]. Recently, the spin-coating technique has been used to build up model films [10]; however, there lack studies on EW films prepared by the dipping technique. This approach is based on the spontaneous deposition of molecules onto a substrate from a solution and can offer a better control of experimental conditions for studies of EW films of plants. Furthermore, it is a spontaneous process of film formation like that found in plants [11]. The aim of this study was to prepare dipping films from epicuticular wax and use them as models for studies of EW films of plants. In these systems, we have investigated the behavior of surface morphology (in terms of pores and aggregates) as a function of the number of layers or temperature. The hydrophobicity of the films was examined with the change of the number of layers by wetting contact angle analysis. A deposition model, called ballistic deposition model, was employed to explain the origin of the pores from aggregates. From our results, it was possible to gain insight on the cuticular water permeability, which plays a key role in the water transport of plant EW films.

## 2 Materials and methods

**Fig. 1:**
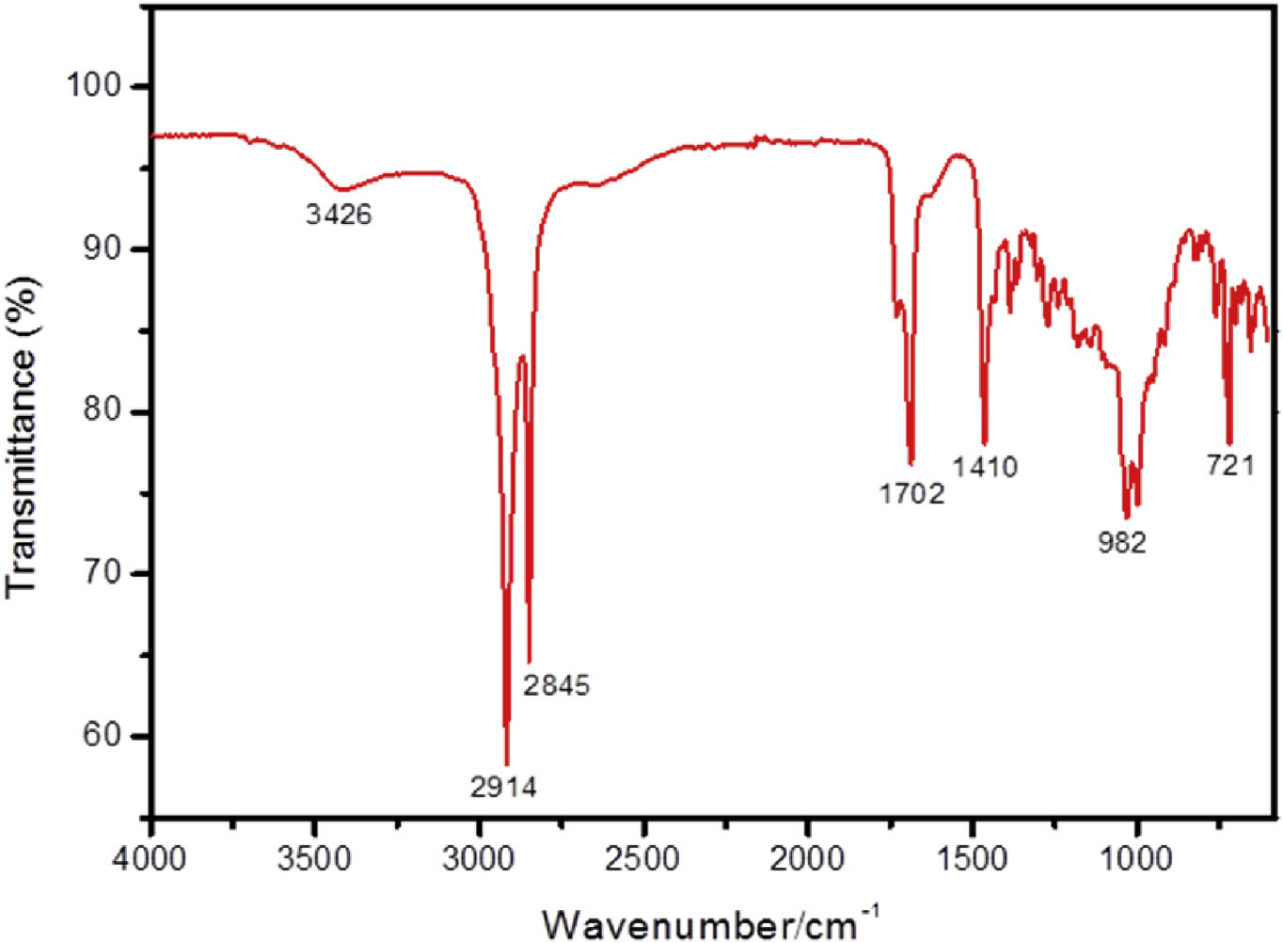
FTIR spectrum for epicuticular wax powder from *Vitis vinífera*.

Epicuticular wax (EW) was extracted from red globe grapes (*Vitis vinifera*), which were purchased at a local market. Waxes were extracted by dipping the grapes in chloroform for 10 min at room temperature (23 °C) [10]. Then, the material was filtered using a filter paper. Before usage, the powder obtained was stored in a dissector for at least one week to decrease the residual solvent. The chemical composition of the EW obtained was determined using a FTIR spectrophotometer (Perkin Elmer FTIR, Spectrometer 100, USA). The vibrational spectrum (Fig. 1) revealed similar vibration bands found in typical epicuticular wax of plants, which are attributed to a mix of alkanes, alcohols, aldehydes, fatty acids, esters, triterpenoids, and hydroxycinnamic acid derivatives [12]. To perform all experiments, a stock solution of EW and chloroform (5 mg/mL) was prepared.

The experiment of growth uniformity was performed by dipping a quartz slide into the EW solution for 60 s, drying, and then obtaining an UV-vis spectrum to determine the maximum value of absorbance at 195 nm. This value is directly proportional to the amount of EW molecules deposited during film formation [13]. For this experiment, an UV-visible spectrometer (Thermolab, Genesys 10, USA) was used. The experiment to study the influence of the number of layers on surface morphology was performed with films of 1, 5, 10, 15, and 20 layers deposited sequentially using an immersion time of 60 s for each one. To avoid an effect of changing concentration, each film was made in triplicate using 10 mL of solution, which was discarded after the immersion of film. The analysis of the surface morphologies of the films was performed with a light microscopic (Celestron, LCD digital microscope 44340, USA) and an atomic force microscope (EasyScan II, NanoSurf Instruments, Switzerland). AFM images were acquired using tapping mode (512 × 512 pixels) with a scan area of 2 μm × 2 μm under ambient condition.

The influence of the number of layers on the hydrophobicity of the films was investigated using an in-house wetting contact angle analyzer described in the literature [14]. The wetting contact angles were measured using purified water droplets (volume of 3 μL), which were gently placed onto the film surfaces in ambient conditions. Six measurements were taken at different locations on each film, and the uncertainty was represented by the standard deviation of sample. The influence of temperature on the morphology of the films was examined by dipping a quartz slide into a solution heated by a thermal bath (SOLAB, SL 152, Brazil) at different temperatures (10, 15, 20, 30, and 40 °C). The analysis of the surface morphology of the films was performed only with the light microscope described previously. For the experiments of the influence of the number of layers or temperature on the surface morphology of the films, counting of the pores of the films was performed using the ImageJ software [15]. The count was made on images with 2048×1536 pixels in size. The steps used for the processing and analysis of images were subtraction of background, binarization and then counting of particles.

## 3 Results and discussion

### 3.1 Growth of the films

**Fig. 2:**
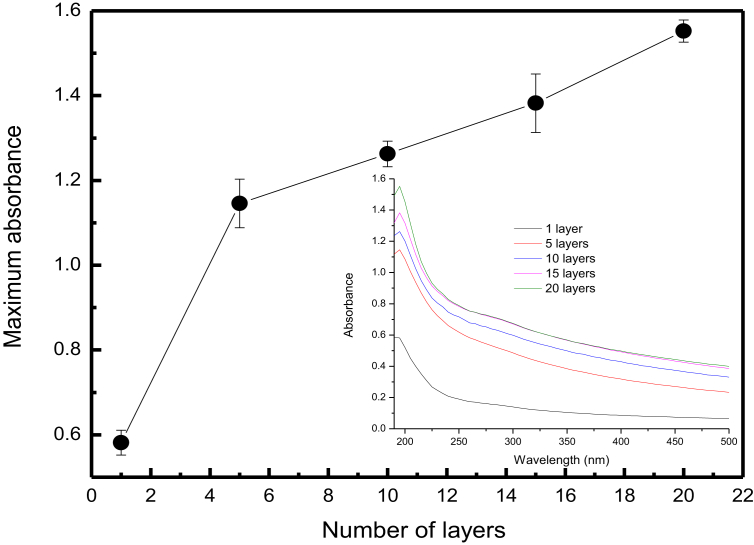
Maximum absorbance (at 195 nm) versus number of layers for EW films. The immersion time was 60 s. The inset shows UV-vis spectra of EW films used for building the plot.

An important requirement to build a film with high quality is the uniformity of growth, i.e., during its growth, the amount of material deposited is constant [13]. To investigate this, we have performed experiments of maximum absorbance – which is proportional to the adsorbed amount of EW molecules – versus the number of layers. Fig. 2 exhibits a plot of the maximum absorbance (at 195 nm) as a function of the number of layers. Each film was prepared by immersing the substrate for 60 s into EW solution. Two regimes of growth are observed: the first one is associated with the influence of substrate, i.e., a large amount of EW molecules is deposited because the bare substrate offers a large number of sites available for adsorption. Then, a linear deposition is observed indicating that the same amount of EW molecules is deposited for each immersion of film [13]. However, the deposited amount is lower than for the first layers. This occurs due to the presence of a fixed number of sites on the film surface. Therefore, from the fifth layer onwards, the adsorbed amount is constant, suggesting good growth uniformity of the films.

### 3.2 Surface morphology

#### 3.2.1 Influence of the number of layers

**Fig. 3:**
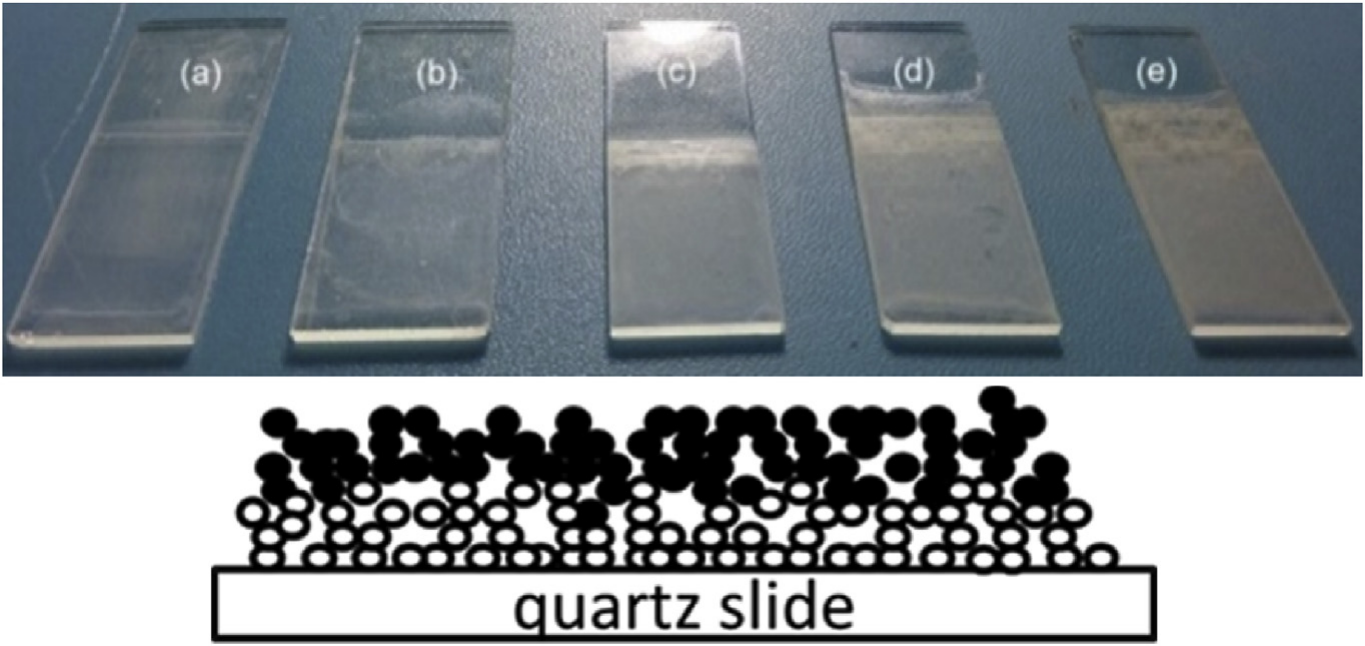
Images of films with different numbers of EW layers: (a) 1, (b) 5, (c) 10, (d) 15, and (e) 20 layers (Top). Squematic diagram of a 2-layer film after ballistic deposition, which can occur during the multilayer film growth. White balls represent the first EW layer and black ones represent the second layer (Down).

**Fig. 4:**
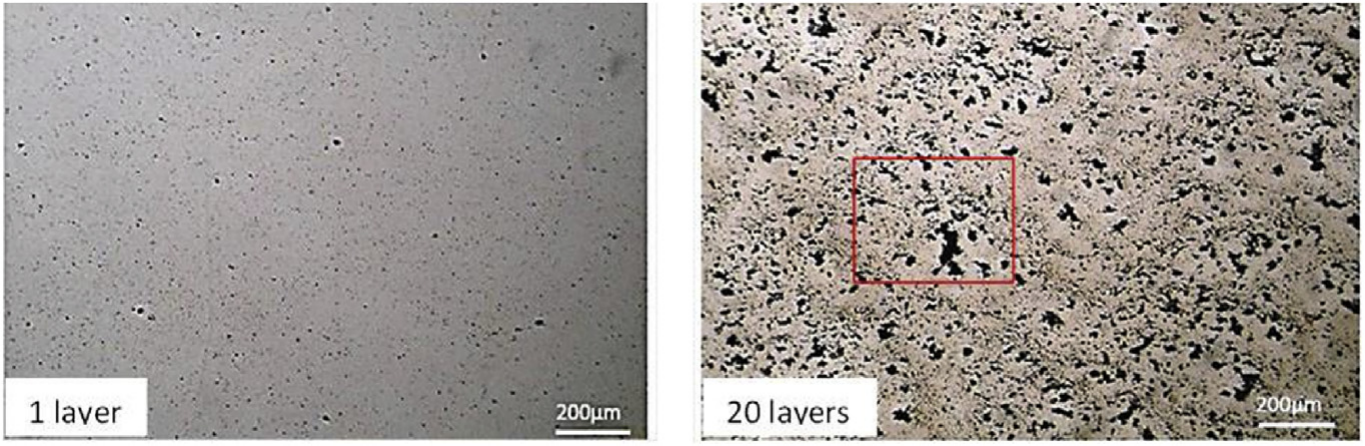
Light microscopy images of epicuticular wax films with two different numbers of layers. The blue square indicates the enlarged area in Fig. 5.

**Fig. 5:**
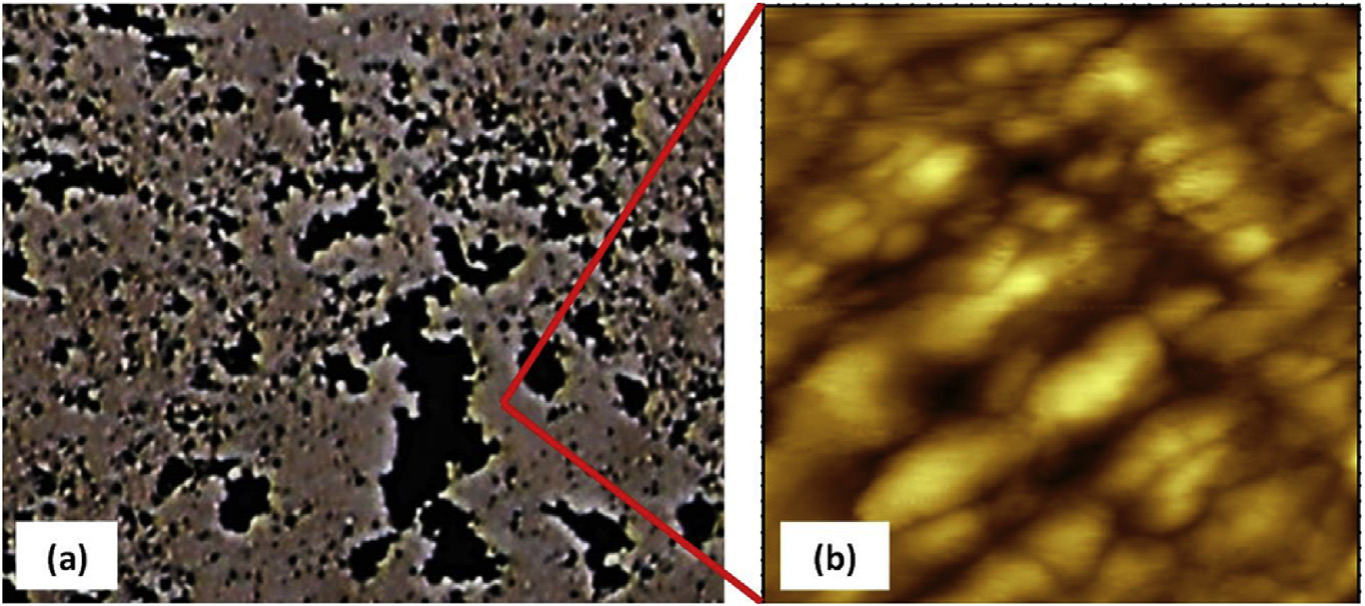
(a) Enlarged image of the 20-layer film from Fig. 4 and (b) AFM image of 20-layer epicuticular wax films with a scan window of 2 μm × 2 μm. The red lines indicate an area scanned by AFM.

Fig. 3 shows images of films used to perform the experiment of film growth. We can observe, at a macroscopic scale, an increase in the adsorbed amount of EW with increasing number of film layers. These films were submitted to light microscopic analysis. For clarity, Fig. 4 exhibits the images corresponding to only two different numbers of layers. To better analyze the film, an enlarged image for the 20-layer film is shown in Fig. 5a. All film surfaces present pores with fractal form. The 1-layer film exhibits a few pores when compared to the 20-layer film (Fig. 4). A formation mechanism of pores from the aggregation of EW molecules can be suggested using the so-called ballistic deposition model (Fig. 3, Down). In this model, several fractal pores are developed during the growth of a fractal surface [16] because particles drop to randomly selected sites of a surface and then stick to the first particle they encounter in their vertical downward motion. This results in a surface formed by pores and aggregates (both with a fractal form) [17]. Therefore, we can assume that the pores in our films result from the growth of fractal aggregates through a ballistic deposition via van der Waals forces. These pores can be considered as the “negatives” of the contours defined by the fractal aggregates.

The previous results suggest that fractal pores formed by fractal aggregates grown by ballistic deposition determine the surface morphology of the films. To confirm this hypothesis, we have performed an AFM analysis of the films. It should be noted that because the AFM tip fell into the pores, images of the fractal aggregates were only obtained after several attempts.

Fig. 5b displays the AFM image of a film with 1-layer, which has already been analyzed by light microscopy (Fig. 4). This image exhibits fractal aggregates with different sizes, which confirms our hypothesis on pores formed by fractal aggregates. Regarding EW films in plants, it is well known they are formed by a self-assembly process via intermolecular forces, in which the EW molecules are deposited onto the plant surface from the cuticle [18]. Our films are also formed by a self-assembly process during the dipping; however, the EW molecules diffuse from solution and then are deposited onto a film surface. Because these films are formed by spontaneous deposition via van der Waals interaction among molecules, such films are feasible models for EW films. It should be noted that studies have found that EW films can exhibit crystalline structures [4, 6]. Then, it is possible that our films also form crystals. To determine whether the EW aggregates (which form our films) have a crystalline structure, more studies need to be made conducted using, for instance, X-ray diffraction analysis.

#### 3.2.2 Pores

**Fig. 6:**
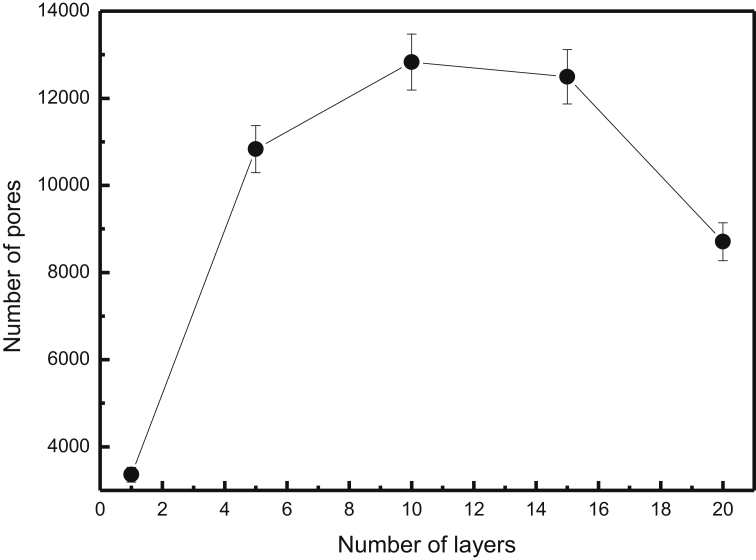
Number of pores versus number of layers. The solid line is a guide to the eyes.

To provide further information on the surface morphology of the films, we have examined the number of pores. Fig. 6 depicts the number of pores as a function of the number of layers from EW at room temperature. Counting of the pores was performed using the films shown in Fig. 3. We note that the number of pores increases with the number of layers up to a maximum value and then decreases. This was expected because the pore sizes increase as the size of aggregates increases during their growth by ballistic deposition. Then, this leads to a decrease of the number of pores because small pores are replaced by large pores.

#### 3.2.3 Influence of the temperature

**Fig. 7:**
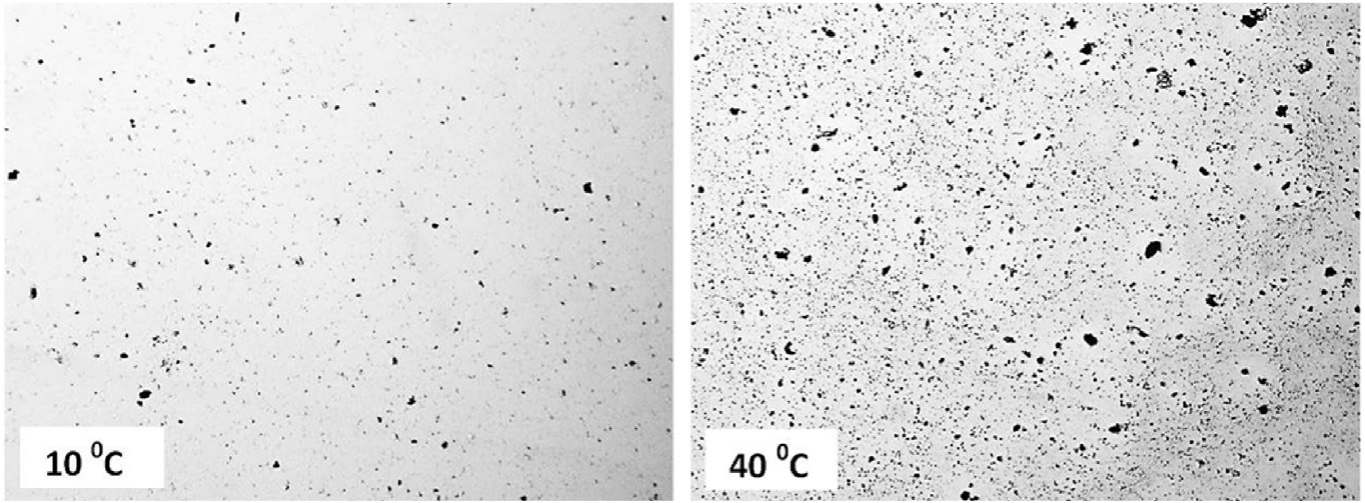
Light microscopy images of 5-layer films at two different temperatures. Magnification of 4×.

**Fig. 8:**
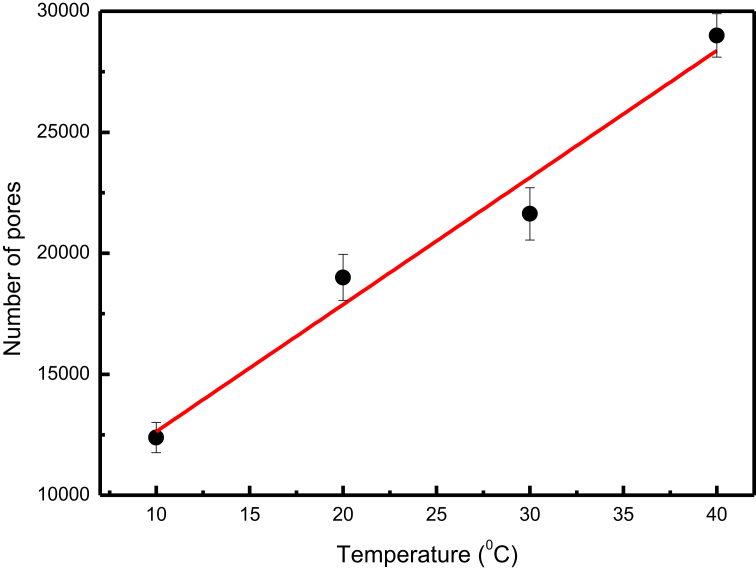
Number of pores versus deposition temperature for 5-layer films. The linear fitting (red straight line) is shown.

**Fig. 9:**
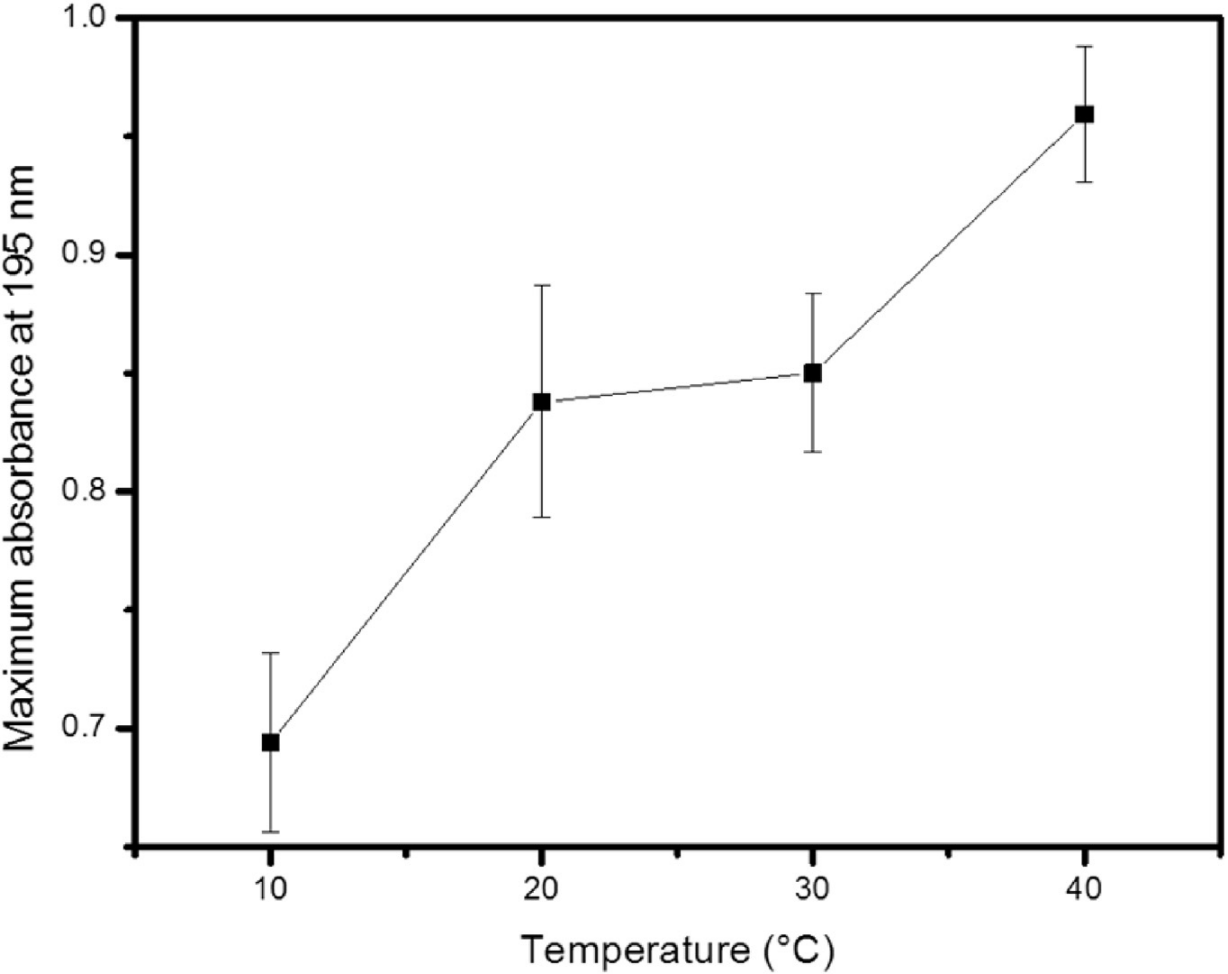
Absorbance versus temperature for a 5-layer film of epicuticular wax.

The water transport phenomenon – which can be characterized by the behavior of cuticular water permeability of EW films in plants – is significantly affected by the environment temperature [19, 20]. Assuming that the surface morphology can affect the permeability via modification of the number and size of pores, we have performed a study of the surface morphology of the films as a function of temperature. Fig. 7 shows representative images obtained from light microscopy for 5-layer films deposited at two different temperatures where several pores are observed (dark areas). We note that the number (Fig. 8) and size of pores (Fig. 7) increase with increasing temperature. At 10 °C, the small number and size of pores are associated with the small number of deposited molecules due to the low mobility of EW molecules. This hypothesis was confirmed by an experiment, which demonstrated that the absorbance – which is assumed proportional to the deposited amount – increases from 10 °C (Fig. 9). In the range from 20 to 40 °C, the number and pore size increase because the ballistic deposition is favored by the sufficient mobility of EW molecules imparted by the suitable temperature. It should be noted that experiments with a range larger than 10–40 °C do not show conclusive results.

For EW films in plants, it has been found that the cuticular water permeability increases with temperature [19]. Considering that the chemical composition does not change, this result suggests that a structural change occurs during the formation of an EW film in response to temperature, leading to the change of the cuticular water permeability. From the insights of our results, we can propose that the cuticular water permeability is determined by the pores, suggesting that at a low enough temperature, the number of pores of the EW film in plants decreases leading to a low permeability, whereas at a high enough temperature, the number of pores increases leading to a high permeability. In another study, it has been found that the cuticular water permeability of plants is independent of the thickness of EW film [18].

In fact, according to our hypothesis in which the pores in the films determine the cuticular water permeability, it is expected that the variation of the thickness does not affect the permeability because it depends only on the pore properties. Experiments to study the water permeability of our films will be carried out to clarify the behavior of the cuticular water permeability of EW films. We should emphasize that an experiment of wettability as a function of temperature was also performed; however, it did not provide reliable results.

### 3.3 Hydrophobicity

**Fig. 10:**
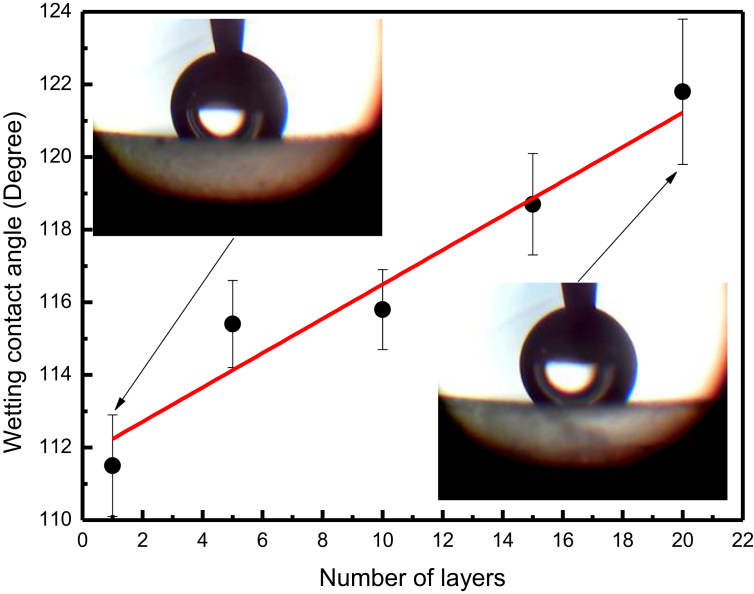
Wetting contact angle versus number of epicuticular wax layers. Error bars represent the sample standard deviations. The insets show illustrative droplets of measurements. The linear fitting (red straight line) is shown.

The hydrophobicity exhibited by EW films of plants is a property essential for these systems because it allows them to repel environmental water [21]. Therefore, hydrophobicity contributes to the control of cuticular water permeability across a plant interface. With this in mind, we have analyzed the behavior of hydrophobicity of our films when the temperature is changed. Fig. 8 shows the wetting contact angle – which characterizes the hydrophobicity – as a function of layers number. We note that the angle increases linearly with increasing layers number in the range of 110 to 120°. It has been found that the wettability of a surface is determined by its chemical composition and surface morphology [22]. Therefore, the increase of irregularities (pores and aggregates) during the film growth (Figs. 4 and 5) can lead to an increase in hydrophobicity observed in Fig. 10. In addition, it is well established that hydrophobic surfaces are those with a wetting contact angle >90° and <150° [14]. Thus, we can classify our films as hydrophobic.

The results of surface morphology indicate an increase in the number of pores with increasing number of layers, suggesting that in the case of a plant, the cuticular water permeability increases as well. On the other hand, the results for the behavior of hydrophobicity (Fig. 10) appear to indicate a decrease of permeability because the higher the hydrophobicity, the higher the water repellence of a plant. Such apparent contradiction can be overcome assuming that the cuticular water transport occurs from the plant to the environment. Therefore, the plant could increase the water outlet, increasing the pore sizes and inhibiting and decreasing its entry at the same time. In addition, it is important to note that the water diffusing from the plant to the environment interacts with the internal film surface that is in contact with the cuticle, forming a film/cuticle interface. Such a surface has a surface morphology and hydrophobicity different from the external surface used for the wetting contact angle analysis, which is performed at the film/air interface.

## 4 Conclusion

Models for EW films of plants were prepared for the first time using dipping films from epicuticular wax. These models were investigated from the viewpoint of the properties of surface morphology and hydrophobicity. Because of the influence of the substrate, it was found that the films presented growth uniformity from the fifth layer of EW onwards with a deposition rate lower than that found in the first four layers. The influence of the number of layers or temperature on surface morphology and wettability of the films was investigated. Our results revealed that pores were produced by aggregates that formed the surface morphology of the films. By analyzing the number of pores, we have noted that their number increased with increasing number of layers, which suggested a ballistic deposition of EW molecules. In addition, the number of pores increased with increasing temperature. Such a result indicated an explanation – based on pores – for the results reported in the literature on the temperature dependence of cuticular water permeability. Concerning the hydrophobicity of the films, we found that it increased with an increasing number of layers. This was associated with the increase of irregularities on the film surfaces, which was consistent with the increase of the aggregate size observed. Dipping films could be an alternative model for studies of EW films found in plants because they have controlled growth and are suitable geometrical forms for the application of experimental techniques. Finally, our findings could contribute to the elucidation of water transport in plants, which has a deep impact in the field of food conservation.

## Declarations

### Author contribution statement

Josmary Silva, Marco Faria, Kevin Santos, Marcos Sousa, Nara de Souza: Conceived and designed the experiments; Performed the experiments; Analyzed and interpreted the data; Contributed reagents, materials, analysis tools or data; Wrote the paper.

### Funding statement

This work was supported by CNPq and CAPES. Marco Faria, Kevin Santos, Marcos Sousa acknowledge CAPES for the scholarship.

### Competing interest statement

The authors declare no conflict of interest.

### Additional information

No additional information is available for this paper.
